# Dynamic Load Balancing of Software-Defined Networking Based on Genetic-Ant Colony Optimization

**DOI:** 10.3390/s19020311

**Published:** 2019-01-14

**Authors:** Hai Xue, Kyung Tae Kim, Hee Yong Youn

**Affiliations:** 1 Department of Electronic, Electrical and Computer Engineering, Sungkyunkwan University, (16419) 2066, Seobu-Ro, Jangan-Gu, Suwon-Si, Gyeonggi-Do, Korea; xuehai@skku.edu; 2 College of Software, Sungkyunkwan University, (16419) 2066, Seobu-Ro, Jangan-Gu, Suwon-Si, Gyeonggi-Do, Korea; kyungtaekim76@gmail.com

**Keywords:** load balancing, Software Defined Networking, genetic algorithm, Ant Colony Optimization, genetic-Ant Colony Optimization

## Abstract

Load Balancing (LB) is one of the most important tasks required to maximize network performance, scalability and robustness. Nowadays, with the emergence of Software-Defined Networking (SDN), LB for SDN has become a very important issue. SDN decouples the control plane from the data forwarding plane to implement centralized control of the whole network. LB assigns the network traffic to the resources in such a way that no one resource is overloaded and therefore the overall performance is maximized. The Ant Colony Optimization (ACO) algorithm has been recognized to be effective for LB of SDN among several existing optimization algorithms. The convergence latency and searching optimal solution are the key criteria of ACO. In this paper, a novel dynamic LB scheme that integrates genetic algorithm (GA) with ACO for further enhancing the performance of SDN is proposed. It capitalizes the merit of fast global search of GA and efficient search of an optimal solution of ACO. Computer simulation results show that the proposed scheme substantially improves the Round Robin and ACO algorithm in terms of the rate of searching optimal path, round trip time, and packet loss rate.

## 1. Introduction

The traditional Internet Protocol (IP) networks need to be enhanced to efficiently deal with huge volume of network traffic generated due to the rapid growth of Internet of Things (IoT). As an outcome of the search for an efficient solution for this issue, a new type of networking paradigm called Software-Defined Networking (SDN) has been proposed [1]. SDN changes the way of traditional network management by breaking the vertical integration of network components, separating the network control logic from the underlying routers and switches, promoting (logical) centralization of network control, and introducing the ability of programming the network operation. OpenFlow is the most widely used communication protocol between the controller plane and switch plane in SDN.

As aforementioned, SDN is quite different from the traditional IP network, and several issues need to be resolved including data forwarding, load balancing (LB), energy management, and so on. In SDN, flow tables are widely used of which entry consists of some fields including the header, counter, and action. When a packet arrives at a switch, lookup of the flow entries is carried out. The header and counter of a network flow are updated when it is changed for some reason such as LB or rerouting. The switch processes the data flow based on the header information and preset rules to control the network traffic. The flow control is based on the algorithm employed to balance the traffic loads [1]. Due to its inherent versatile nature of SDN, dynamic LB is very important for the centralized controller of SDN.

There exist various studies on LB of SDN. Kang et al. [2] presented a scheme on how to use the genetic algorithm (GA) for solving the LB problem of SDN. Even though it is an innovative approach, the classical GA is highly time-consuming. There are also studies adopting Ant Colony Optimization (ACO) for LB [3,4,5]. In Li et al. [3], a traffic-engineering framework was proposed which contains a heuristic algorithm layer and meta-layer based on machine learning. For the traffic engineering, the heuristic algorithm layer is trained by the meta-layer to find an optimal path after training. Then, dynamic routing occurs via the optimal path, resulting in efficient network operation. ACO was also employed in QoE-Centric flow routing [4], which was shown to be better than Shortest Path Routing (SPR). In Lin et al. [5], ACO was combined with job classification for multi-controllers, where job classification distinguishes central controllers from sub-controllers. Even though the basic ACO algorithm renders good results, a lot of computations are needed before it is fully operational. As with the typical optimization problems, the solution may take a long time to converge or lead to the local optimum. It is also not easy to be deployed to the network when the operation scenario changes.

In this paper a novel approach for LB of SDN is proposed which combines GA with ACO, called Genetic-Ant Colony Optimization (G-ACO). The existing scheme based on the ACO algorithm employs the positive feedback mechanism for updating the path information of the flows when they are forwarded. However, this may result in a local optimal solution and improper selection of the path. The random selection policy can be employed to avoid the local optimal solution, but it does not guarantee even suboptimal solution. These issues limit the performance of ACO in the path selection of SDN. The trade-off between local optimal solution and random selection is still an issue with ACO. The GA is thus adopted with ACO to alleviate the problem and enhance the LB of SDN. The GA is applied in the second stage of the search to effectively reduce the search space, whereupon the ACO algorithm can efficiently find the paths of the flows for LB. Computer simulation reveals that the proposed scheme substantially improves the roundtrip time (RTT) and packet delivery ratio compared to the Round Robin (RR) and ACO algorithm by effectively achieving the LB.

The rest of the paper is structured as follows. Section 2 provides an overview of the ACO and GA, and LB for SDN. In Section 3 a new method for dynamic LB of SDN is proposed. Also, an analytic model of the proposed scheme is presented. Section 4 gives the performance evaluation of the proposed scheme with the controller, OpenDayLight, and switch emulator, Mininet. Finally, Section 5 concludes the paper and outlines future research direction.

## 2. Related Work

In this section SDN, GA, ACO, and LB are briefly discussed. LB is a crucial problem with SDN, while GA and ACO are the algorithms which are adopted in the proposed scheme for solving the problem.

### 2.1. SDN

SDN is a new technology in the field of computer networking, which is presently receiving a great deal of attention. It originated from a project in academia [5]. In SDN, the network control making decisions on traffic routing (control plane) is decoupled from the part forwarding the traffic to the destination (data plane). The network is directly programmable, and the infrastructure is allowed to be abstracted for the applications and network services. The experts and vendors claim that this greatly simplifies the networking task [6]. Here, balancing the traffic load of the switches is a very important issue. 

LB has been extensively studied for traditional IP network. Recently, some studies were conducted on SDN. Since SDN decouples the control plane and data plane of the network, LB between the controller and switches directly affects the stability of the whole network. The controller running on the control plane controls data forwarding by managing the flow tables of switches. The flow table consists of the flow entry, and each flow entry mainly consists of six fields as listed in Table 1 [7].

**Figure 1:**
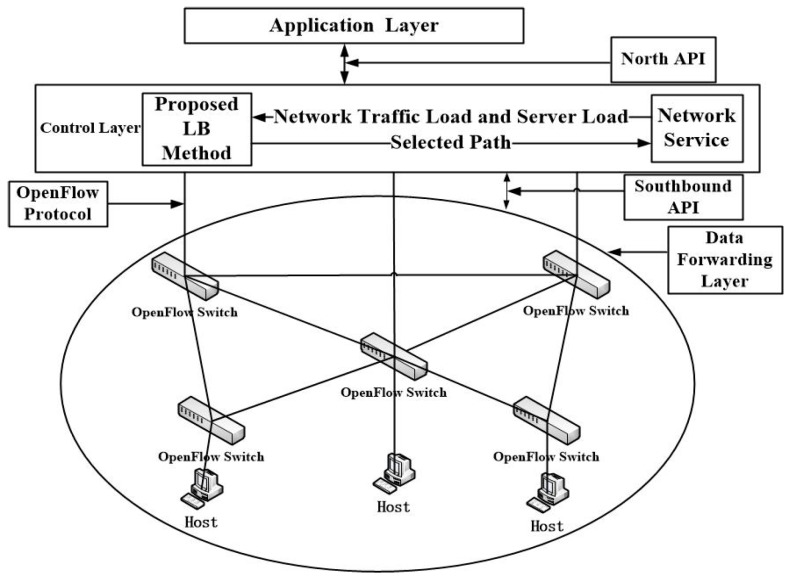
The structure of SDN based on the OpenFlow protocol.

Among several protocols proposed for SDN, the OpenFlow protocol [8] is the most commonly used one for the implementation of SDN. The main feature of OpenFlow is the programmability. By programming the controller, one can easily achieve the required network functionality. There is a total of three layers in the entire infrastructure of SDN. On top of the control plane is the application layer and below is the data forwarding layer. The structure of SDN based on the OpenFlow protocol is shown in Figure 1.

### 2.2. ACO

The ACO algorithm employs a probabilistic approach for solving the target problem such as GA, simulated annealing, and so on. All such algorithms are based on some heuristics. The ACO algorithm was inspired by the behavior of ants. Initially, all the ants wander aimlessly, but they return the nest after laying down pheromone on the trails while searching for food. Other ants then can follow the pheromone trails instead of travelling randomly. This approach has been adopted to solve the problem of computer networking such as finding the best path in the network. There are various schemes based on the ACO algorithm such as AntNet, AntHocNet, HopNet and Stigmetry, which can be applied to solve the routing problem for computer network [9,10]. 

The ACO algorithm works in two steps. In the first step, the preceding ants discover new possible routes as well as gather information on the existing paths, which are referred to as forward update. If one of them successfully reaches the destination node, in the second step, some ants are sent back to the source node along the path previously explored by the preceding ant. During the backward trip, the routing tables of the nodes along the path are updated called backward update. With SDN, the forward update and backward update can be eliminated since the controller has the global view of the entire network [11].

### 2.3. GA

As aforementioned, GA is also a heuristic algorithm based on the evolutionary steps of natural selection and genetics. It is commonly used to generate high-quality solutions for the optimization and search problems using bio-inspired operators such as mutation, crossover, and selection [12]. Some researchers explored an intelligent exploitation of random search used to solve the optimization problem involving global search of a large space. Generally, there exist five steps in GA for solving a problem; initial population, fitness function, selection operation, crossover operation, and mutation operation. In the proposed scheme, the five steps are also adopted as a supplement to ACO in the second stage of the search of the path. It applies selection, intercross and mutation on the solution of each generation to broaden the search domain and reduce the search time of optimal path.

### 2.4. LB of SDN

There exist two ways of classifying the LB approach of traditional network. One is based on the object to which LB is applied; hardware LB and software LB. With hardware LB, LB is applied to individual hardware device, and thus its effect on the network operation is more direct and faster than software LB. However, it is costlier due to the involvement of hardware implementation. Another way of classification is static LB and dynamic LB, determined based on the adaptability of the traffic management policy. With static LB, the performance parameters are analyzed at the start of the network operation, and then the LB policy is determined based on that. It is easy to deploy and not costly, but the performance is low. With dynamic LB, the LB policy is dynamically updated based on the current condition of the network to achieve higher performance. However, it usually involves several steps of operations and the cost is higher. Due to the versatile characteristics of SDN, the software LB and dynamic LB policy are adopted in the proposed scheme.

Various LB algorithms have been developed for evenly distributing the traffic to the nodes in the network including the RR algorithm, Weighted Round Robin (WRR), Least-Connection (LC), Weighted Least-Connections (WLC), Fastest Response-Time (FRT) algorithm, and so on. With the RR algorithm, a round link table is managed, which holds the information on the nodes of the network. The tasks are forwarded to each node by circular turns so that each node gets the task equally likely. This algorithm is easy to implement, but it does not consider the condition of the nodes and thus congestion may easily occur. Unlike the RR algorithm, the WRR algorithm assigns a weight value to each node which is determined by the specification parameter of the nodes. A large weight means higher processing capability. WRR is obviously more effective than RR.

The LC algorithm assigns a new connection request to the node of least connection since the load condition of a node is estimated based on the number of connections. The device managing LB needs to keep and update the number whenever a new connection is made or one is disconnected. The WLC algorithm uses the weight with the LC algorithm. The node of higher weight is assigned more connections. The FRT algorithm analyzes the load condition of a device by sending a probe request to it (for example ping). Here, the server responding to the probe fast is deemed to be less busy. Since it is not the response time to an actual task but a probe, it may not be able to deliver the real load condition. We next present the proposed scheme combining GA with ACO, the G-ACO scheme, which capitalizes the key properties of SDN for LB.

## 3. The Proposed Scheme

In this section, first, the way the basic ACO algorithm is used for the LB of SDN is introduced along with its shortcomings. Then, the proposed LB scheme based on G-ACO is presented. 

### 3.1. LB with ACO

**Figure 2:**
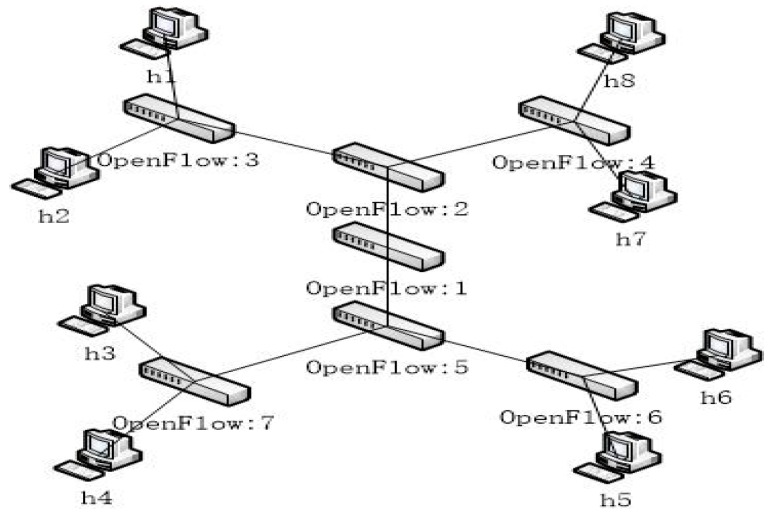
The test topology.

In order to illustrate the concept of ACO employed in the proposed scheme, the topology shown in Figure 2 is used. Each ant in the ant-world can be viewed as a packet transmitted in the network, and it is started randomly at any node of the topology.

Assume that *m* and *n* are the total number of ants and switches, respectively, and *b_i_*(*t*) represents the number of ants in Switch-*i*, *S_i_*, at time *t*. Then,

(1) $$m=\overset{n}{\underset{i=1}{\sum }}b_{i}\left(t\right)$$

Each ant, *A_k_* (*k* = 1,2, …, *m*), bears a taboo table to avoid visiting the same switch again, which holds the switches already passed by. It also calculates the state transition probability between two switches based on the remained pheromone on each path. They select the next switch to visit depending on the calculated probability. Denoting *P_ij_^k^*(*t*) as the probability of *A_k_* to visit *S_j_* from *S_i_* [13], then:

(2) $$P_{ij}^{k}=\{\begin{array}{c} \frac{{\left[\tau_{ij}\left(t\right)\right]}^{\alpha }\cdot \eta_{ij}{}^{\beta }}{\sum_{a_{k}}{\left[\tau_{ij}\left(t\right)\right]}^{\alpha }\;\cdot \eta_{ij}{}^{\beta }]},\;j\in a_{k}\\ \;0\;\mathrm{Others} \end{array}$$

Here *a_k_*represents the switches available to be selected when *A_k_* is in *S_i_*. *τ_ij_*(*t*) denotes the total pheromone laid on the path from *S_i_* to *S_j_*. *τ_ij_*(0) = *δ* indicates that the amount of pheromones on path is initially *δ*. *α* and *β* are the weight of the remaining pheromone and path distance in making the selection, respectively. *η_ij_* is a heuristic function defined as:

(3) $$\eta_{\mathrm{ij}}=\frac{1}{d_{\mathrm{ij}}}$$

Here, *d_ij_* represents the distance between *S_i_* and *S_j_*. Therefore, the probability of visiting *S_j_* from *S_i_* becomes higher as *d_ij_* gets smaller. The two issues with the current mechanism of ACO in selecting the next switch are as follows.

- If *α* is small, it depends mainly on the value of *d_ij_*.
- If *β* is small, it is selected randomly.

The first issue may cause the local optimal solution, while the second one does not guarantee finding any good solution. To resolve the issue of too much remaining pheromone, an update algorithm was proposed considering the behavior of real ants in nature [14]. Here, the pheromone of a path is updated when an ant passes a switch (partial renewal) or whole path (global renewal), and the update rule is as follows.

(4) $$\tau_{\mathrm{ij}}\left(t+n)=(1-\rho )\;*\;\tau_{\mathrm{ij}}\;(t)\;+\;\Delta \tau_{\mathrm{ij}}(t\right)$$

(5) $${\Delta \tau }_{\mathrm{ij}}\left(t\right)=\overset{m}{\underset{k=1}{\sum }}{\Delta \tau }_{\mathrm{ij}}^{k}\left(t\right)$$

Here *ρ* is the percentage of pheromone volatilization on the path, and thus (1−*ρ*) represents the portion of remaining pheromone. Δ*τ_ij_^k^*(*t*) is the amount of pheromone increased by *A_k_* on the path from *S_i_* to *S_j_* in one cycle. Δ*τ_ij_^k^*(0) is 0 as it represents the initial state of each path.

In the process of searching an optimal path with ACO, each ant selects the next switch based on the probability of Equation (2). Meanwhile, a positive feedback mechanism is adopted to assign more weight to the current optimal path. In other words, the information on the traversed path is given more weight after each iteration. This approach can easily cause local optimum. If the random selection policy is employed to avoid local optimum, even a good solution is not guaranteed to be found or it may take long time. These issues are resolved by the proposed scheme explained next.

### 3.2. The Proposed G-ACO Scheme

Delay time and packet loss rate are the two criteria evaluating the Quality of Service (QoS) of the network [15]. To deal with the limitation of the ACO algorithm applied to the SDN for LB, the G-ACO algorithm is proposed. As in the typical GA, it involves selection, crossover, and mutation operation. The motivation is to enhance the rate of convergence to optimal LB and the capability of finding the global optimum. The delay time in sending packets and the packet loss rate will then be decreased. It is known that GA is effective for the global search of a large space. However, feedback information cannot be used and a lot of redundant iterations occur with a certain search space. The efficiency of finding the solution is also very low. On the other hand, ACO employs a positive feedback mechanism. However, in the second stage, the searching speed is very low due to the limited pheromone on the paths. The proposed G-ACO algorithm capitalizes the merit of the GA and ACO such that GA is used to properly distribute the pheromone and then the ACO algorithm is used to seek the solution. It lets the two algorithms complement each other for effective LB in SDN. The larger the network and the longer it has been operated, the more the proposed G-ACO scheme will decrease the RTT and packet loss rate using the accumulated information compared to the existing schemes. The steps of G-ACO algorithm are as follows. 

• Step 1. Initial population

With G-ACO, the path is encoded for searching the path which involves integral number. For example, assume that a packet reaches a host in one iteration through several switches, which are *S*_7_, *S*_5_, *S*_1_, *S*_4_, and *S*_8_. Then, the code of the path of this packet is (7,5,1,4,8). 

One path is generated with each ant in one iteration. Since there are *m* ants, *m* paths are obtained, which are selected as the initial population.

• Step 2. Fitness function 

There are several factors that need to be considered for deciding the fitness function of GA, including the length of path, energy consumed for receiving or sending a packet in each switch, energy status of the whole network, and so on. In this paper the length of the path is considered as the primary factor. The fitness function for path-*p* is then as follows:

(6) f(p)=W×l(p)

Here *W* is the weight value of the length and *l*(*p*) is the length of the path. Note that the path of smallest *f*(*p*) is selected. 

• Step 3. Selection

In the selection process, the code of a path is obtained after each iteration of search. The load is calculated by the fitness function, and then the optimal one is selected for the next iteration. After several selection operations, the pheromone on the optimal path will be larger than the others, indicating that it will be more likely to be selected. As a result, the speed for searching the optimal path is increased. 

• Step 4. Crossover

In order to avoid the situation of stagnation during the search operation, the crossover operation is adopted. It can expand the search scope and avoid the local optimum. After an iteration of search by ACO, some sub-optimal paths and optimal path might be obtained. Then, the crossover operation is applied to them. The aim of this step is to include more candidate paths leading to the optimal one. 

Here, the crossover operation proposed by [16] is used such that the crossover operation is performed with the predefined crossover probability. The subsequence of the path of the offspring is determined from the subsequence of the paths of the parent. For example, assume that two parents, *P*_1_ and *P*_2_, are given:

(7) $$P_{1}=(9,8|\;7,6,5|\;4,3,2)\;P_{2}\;=\;(2,4|\;7,5,8|\;6,9,3)$$

In order to get the sequence of the offspring by crossover operation, first the cross segment is copied to the sub-generation, *g*_1_ and *g*_2_, as:

(8) $$g_{1}=(**|\;7,6,5|\;***)\;g_{2}\;=\;(**|\;7,5,8|\;***)\;$$

Then, the coincident character sequence needs to be deleted in both *P*_2_ and *g*_1_. For example, ‘7’, ‘6’, ‘5’ of *g*_1_ is deleted from *P*_2_, which leaves (2,4| 8| 9,3) in *P*_2_. After the deletion, the remaining sequence of *P*_2_ is copied to get *g*_1_ = (2,4| 7,6,5| 8,9,3). Similarly, *g*_2_ = (9,6| 7,5,8| 4,3,2) is obtained. 

In this paper *P*_1_ is assumed to be the optimal path, while *P*_2_ is sub-optimal. Procedure 1 of crossover operation is as follows.

**Procedure 1.** Crossover operation of G-ACO1: Assume that the path of *P*_1_ is (*x*_1_, *y*_1_, *z*_1_) and that of *P*_2_ is (*x*_2_, *y*_2_, *z*_2_). The crossover operation occurs with *y*_1_ and *y*_2_, and one of the new paths obtained is *P*_3_: (*x*_1_*, y*_2_*, y*_1_*, z*_1_).2: Deleting the duplicated switch, the new path *P*_3_ is determined. 3: By the same way, another new path *P*_4_ is obtained. 4: Applying the fitness function to *P*_1_, *P*_2_, *P*_3_, *P*_4_, an optimal path is selected

• Step 5. Mutation

Mutation operation is based on the predefined mutation probability. Two points from the paths of the offspring are randomly selected for mutation operation. For example, for the given *g*_1_,

(9) $$g_{1}=(2,4|\;7,6,5|\;8,9,3)$$

After the mutation operation of exchanging ‘5’ and ‘3’, *g*_1_ becomes:

(10) $$g^{\prime }=(2,4|\;7,6,3|\;8,9,5)$$

Procedure 2 of mutation operation is given below.

**Procedure 2.** Mutation operation of G-ACO1: The mutation occurrence is based on the frequency which is defined in the simulation part, and the number of switches in the optimal path is *m*.2: Randomly generate two natural numbers, *n*_1_ and *n*_2_ (*n*_2_ < *n*_1_ < *m*). 3: By exchanging the switch at the location of *n*_1_ and *n*_2_ of the optimal path, *P*_0_, a new path, *P_n_* is obtained. 4: Obtain the fitness of *P*_0_ and *P_n_*, and the one of the smaller value is selected as the optimal path

**Figure 3:**
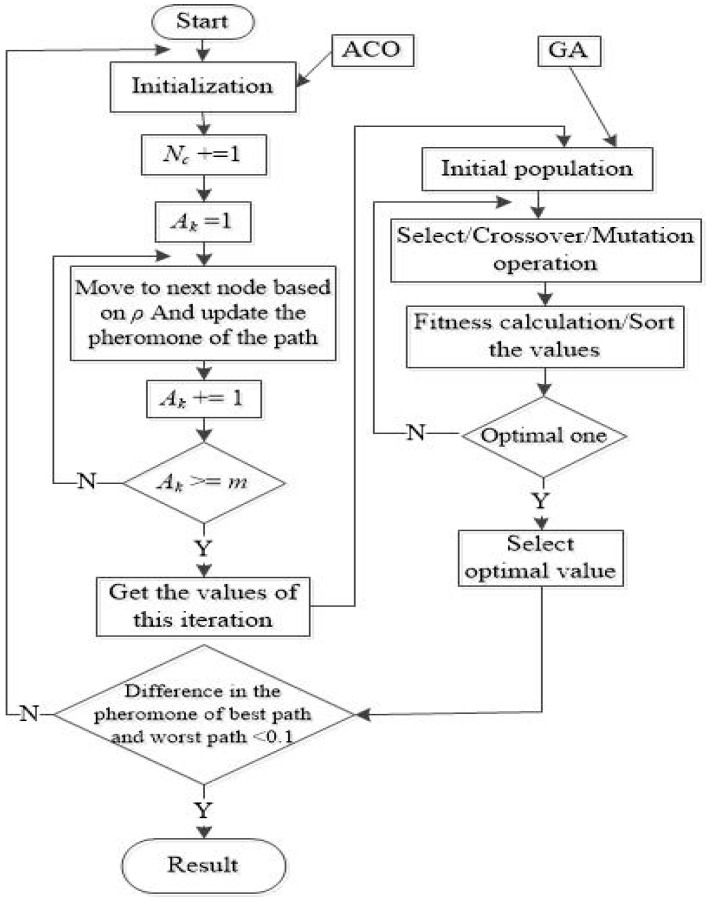
The flowchart of the proposed G-ACO.

After the operations of G-ACO, the optimal path is determined and packets are transmitted along it. The basic steps of the proposed G-ACO are summarized in Procedure 3, and the flowchart of the proposed G-ACO Algorithm is shown in Figure 3.

**Procedure 3.** LB with G-ACO1: *N_c_* + = 1; (Iteration times)2: *A_k_* = 1; 3: *A_k_* selects the next switch based on the calculated probability of Equation (2); meanwhile, update the taboo table and path pheromone. 4: *k* + = 1; 5: If *k* ≤ *m* (the total number of ants), go to Step 3, otherwise execute the GA; 6: Obtain the fitness value, and if the value satisfies the constraint, exits the loop. Otherwise, jump back to Step 1

## 4. Performance Evaluation

In this section an experiment is conducted to evaluate the performance of the proposed scheme. Also, it is compared with the RR and ACO scheme used for LB of SDN to verify its effectiveness. 

### 4.1. Environment of Experiment

There exist separate tools used for the SDN controller and switch as the control plane and data plane are decoupled. In this paper OpenDayLight and Mininet are employed for setting up the controller and switch, respectively. First, a brief introduction of these tools is given.

Mininet is a network emulator creating a network of virtual hosts, switches, controllers, and links. It hosts standard Linux network software, and the switches employ OpenFlow for flexible routing with the SDN. Mininet supports research, development, learning, prototyping, testing, debugging, and many other tasks that could benefit from having a complete experimental network on a laptop or PC [17]. Some key features of Mininet are listed below.

- Supports relevant protocols of SDN such as OpenFlow
- Supports open Python API for developers
- Supports relevant modules of SDN such as Open vSwitch
- Highly scalable
- Developers can customize the topologies as needed
- Supports co-development among several engineers

OpenDayLight [18] is a collaborative open source project hosted by the Linux Foundation. The goal of the project is to accelerate the adoption of SDN and create a solid foundation for Network Functions Virtualization (NFV). It is written in Java, and the main modules of OpenDayLight are as follows:

- Topology Manager: Responsible for the entire network topology
- Forwarding Rule Manager: Manages the actions of entire network by adding, searching, deleting, and updating the flow rules
- Service Abstract Layer: Core module of OpenDayLight, abstracting some parts of the network by southbound interface and supporting application layer
- Host Tracker: Manages the information of the hosts by keeping the IP and MAC address, and establishes and deletes the connections to northbound interface
- Stats Manager: Manages the whole information of the network

In this experiment OpenDayLight and Mininet are installed separately on two PCs. The parameters of the PCs are listed in Table 2.

### 4.2. Test Topology

**Figure 4:**
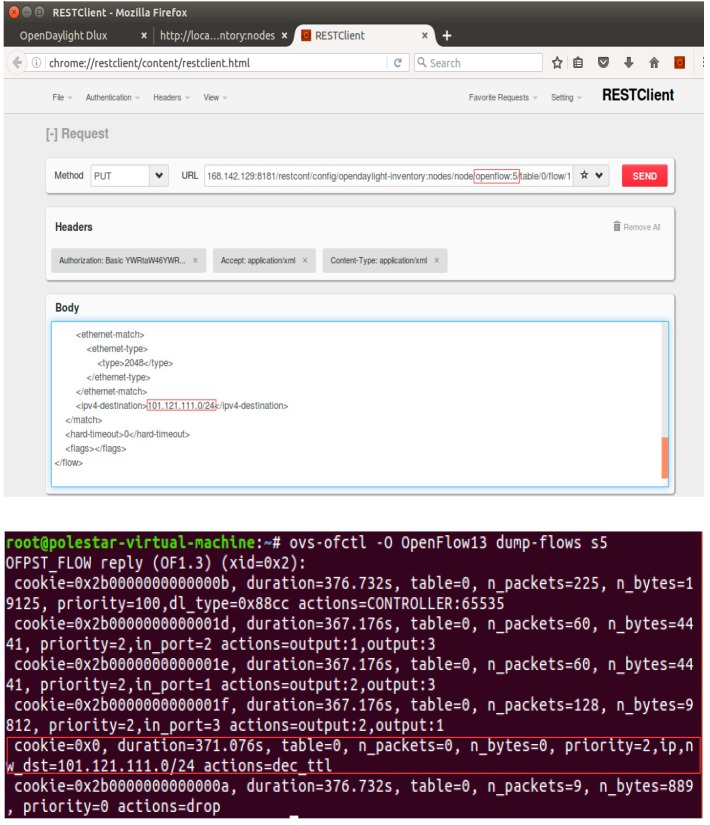
The result of flow table delivery.

At first, some flow tables were sent to the specified switch with the topology of Figure 2 to verify proper connection of OpenDayLight and Mininet. Some flow tables were sent to *S*_5_ (OpenFlow:5), and the flow table delivery was verified as the snapshot shown in Figure 4.

**Figure 5:**
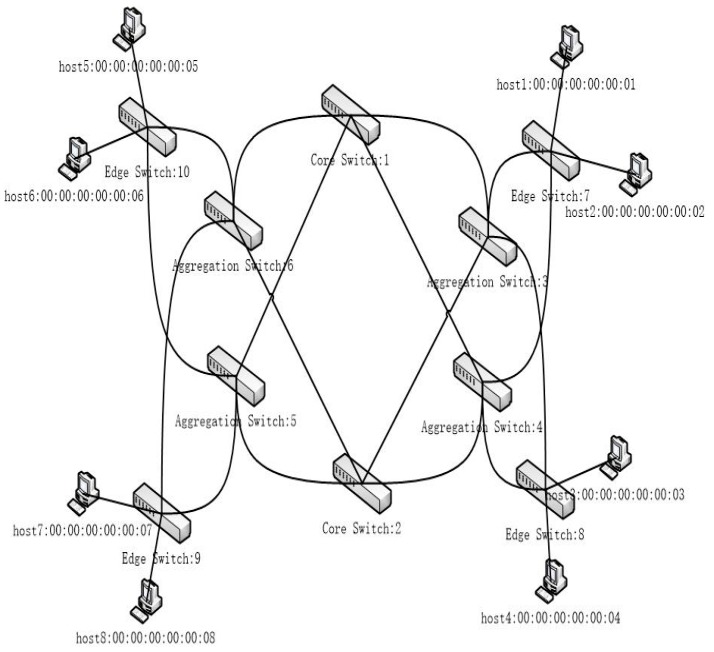
The target fat-tree topology.

A fat-tree topology is adopted to verify the effectiveness of the proposed LB scheme. The fat-tree topology has numerous advantages as elaborated in [19,20], and the target fat-tree topology consisting of 1 controller and 10 switches is shown in Figure 5.

### 4.3. Simulation Results

For fair comparison of the proposed scheme with the existing schemes, the Ant-Cycle method is employed for pheromone update [21]. The update rule and increment of pheromone are based on Equations (4) and (5), respectively. However, Δ*τ_ij_^k^*(*t*) is modified as follows:

(11) $${\Delta \tau }_{ij}^{k}\left(t\right)=\{\begin{array}{c} \frac{Q}{L_{k}}{\mathrm{if}\;A}_{k}{\;\mathrm{moves}\;\mathrm{from}\;S}_{i}{\;\mathrm{to}\;S}_{j}\;\mathrm{in}\;\mathrm{one}\;\mathrm{iteration}\\ 0\;\mathrm{otherwise} \end{array}$$

Here, *L_k_* is the total length of *A_k_* for finishing a round trip. As both the ACO and GA have the characteristics of astringency, the proposed G-ACO scheme may also have similar property. The factor *ρ* in Equation (4) can affect the search capability and the speed of astringency of the whole network. If *ρ* is too large, the volatilization rate of pheromone is high, leading to random selection. On the contrary, if it is too small, the volatilization rate will be low, resulting in local optimum. Therefore, selecting a proper value of *ρ* is very important. In the experiment, *m* (the number of ant) = 6, *α* (pheromone weight) = 1, *β* (distance weight) = 2 and *Q* (constant) = 1, which are determined empirically. The numbers of iteration steps allowing optimal path are listed in Table 3 as *ρ* is varied.

Notice from Table 3 that the number of iterations increases when *ρ* is raised. This implies that the amount of pheromone on a path significantly delays the path selection. On the contrary, when *ρ* is as low as 0.1, the amount of pheromone on the path imposes too much influence on the path selection. Referring to Table 3, the reasonable range for *ρ* seems to be 0.3~0.5, and thus the value of *ρ* is set to be 0.4 in the experiment.

In Equation (2) *α* and *β* are important parameters of G-ACO for an ant to select a path. *α* determines the dependency on the pheromone. If *α* increases, the probability for an ant to follow the path taken by a previous one will be higher. Then, the random nature of path selection will become smaller, leading to local optimum. On the contrary, *β* is the factor for randomness in path selection. If *β* increases, the probability for an ant to randomly select the next path becomes higher. Then, an optimal path may not be found in reasonable amount of time. Therefore, a simulation was carried out to determine a proper value of *α* and *β*. First, the value of *α* and *β* is set empirically. Then, *α* is changed while *β* is fixed. Similarly, *β* is changed while *α* is fixed. The simulation results are shown in Table 4, which show that the number of iterations decreases while *α* and *β* get larger. Here, *α* and *β* are set to 1 and 2, respectively. 

In the proposed G-ACO scheme there is another important factor which is the number of ants, *m*. If it is too small, the search of the whole network will be limited, leading to local optimum. If it is too large, however, the effect of positive feedback diminishes in path selection, resulting in random path selection. Considering the importance of the number of ants, simulation has been carried out again to determine the number. The simulation result is shown in Table 5. Considering the convergence and randomness of G-ACO, *m* is set to 8.

In summary, the parameters are set as *ρ* = 0.4, *α* = 1, *β* = 2, *m* = 8. For the GA, the probability of crossover and mutation are set to be 0.9, and 0.04, respectively. Finally, the convergence condition, which is the difference in the amount of pheromone on the largest and smallest path, is set to 0.1. 

**Figure 6:**
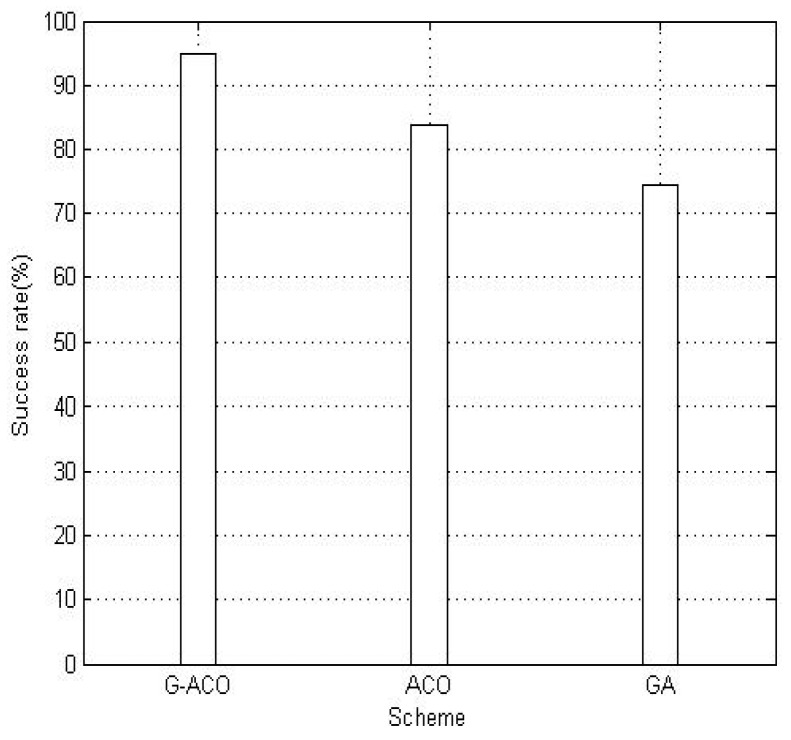
The comparison of success rates.

Figure 6 shows the success rate of finding the optimal path with the proposed G-ACO, ACO and GA scheme. An optimal path from the initial node to the end node can be determined by the calculation of the fitness function. Then, a path is obtained with the respective scheme after each simulation run. By the comparison with the optimal path, it is judged whether it is an optimal path or not. A 10-minute simulation time is executed for each of the three schemes, and the success rates are compared in Figure 6. Observe from the figure that the proposed G-ACO scheme allows an approximately 95% success rate which is much higher than the other two schemes. 

**Figure 7:**
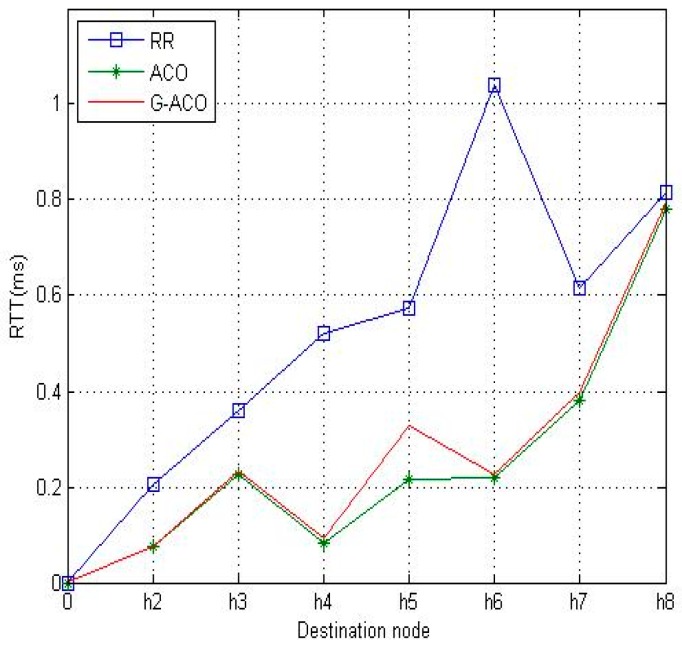
The comparison of RTTs with 5-minute simulation time.

**Figure 8:**
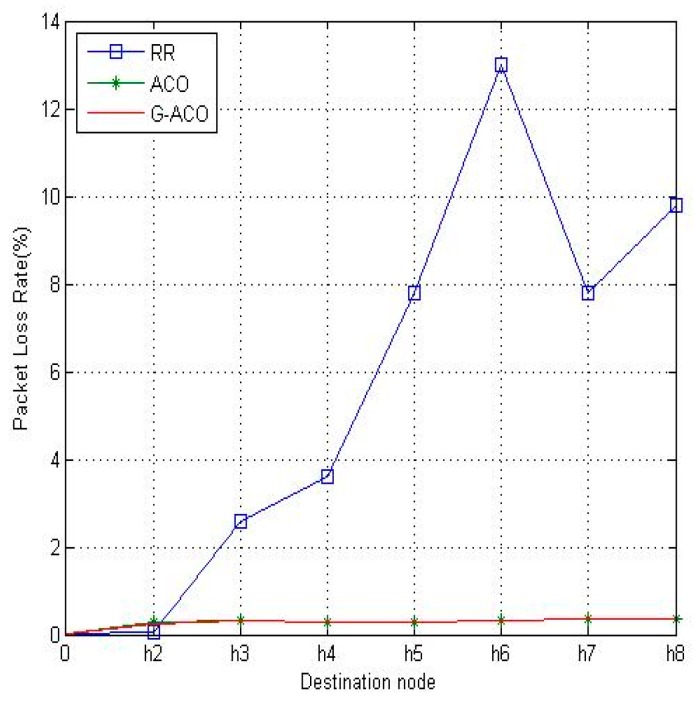
The comparison of packet loss rates.

In the target network, the bandwidth of each load is assumed to be 100 Mbps. The iperf software tool [22] is used for the simulation run for 5 min and 10 min. The RR, ACO and the proposed G-ACO are simulated, RTT and packet loss rate are collected with *h*_1_ of Figure 5 as the source node of the packet. The simulation results with 5-minute simulation time are shown in Figure 7 and Figure 8. Observe from the figures that the RTT and packet loss rate of the proposed G-ACO scheme are almost same as the ACO scheme. When the simulation time is as small as 5 min the computation overhead of the GA in the initial phase is high while the accumulated information required for the path selection with the proposed G-ACO scheme is insufficient. This causes similar performance between G-ACO and ACO.

**Figure 9:**
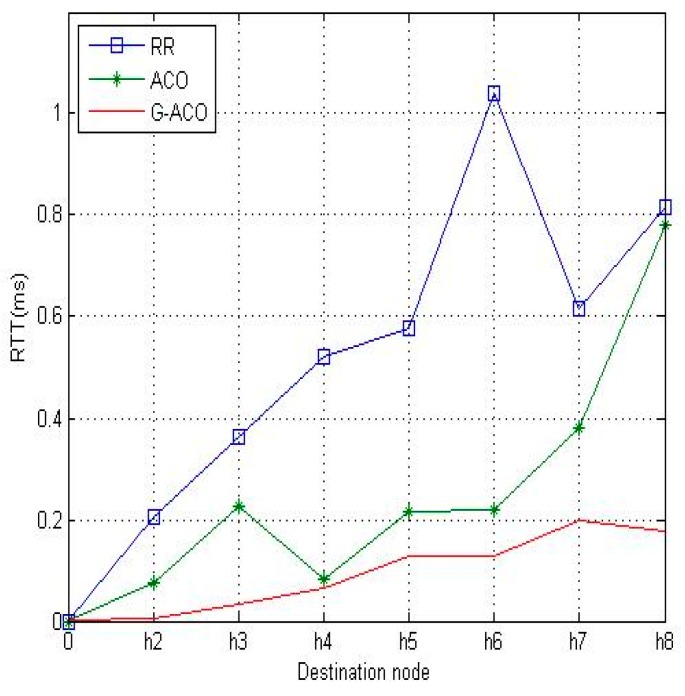
The comparison of RTTs with 10-minute simulation time.

**Figure 10:**
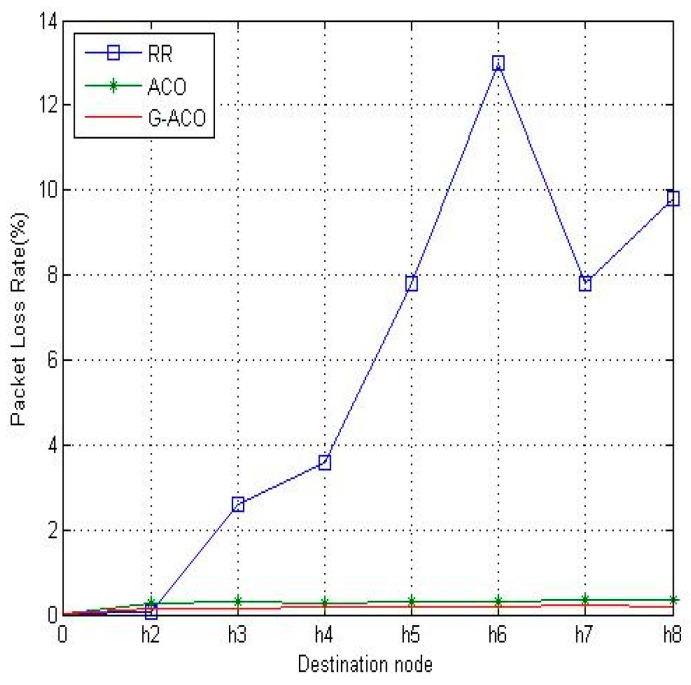
The comparison of packet loss rates.

Figure 9 and Figure 10 are the results with the increased network running time of 10 min. Notice that the proposed G-ACO scheme significantly reduces the RTT and packet loss rate compared to the other two schemes. This is due to the fact that more information can be accumulated as the operation of the network lasts longer. Recall that the proposed G-ACO scheme takes advantages of the merits of both GA and ACO, the fast global search of GA and optimal search of ACO. 

From the simulation results above, it was identified that the proposed G-ACO scheme substantially reduces the RTT and packet loss rate compared to the other two schemes. Notice that the packet loss rate of *h*_1_–*h*_6_ is very high with RR. This is because the load of *h*_1_–*h*_6_ causes congestion on the path. For this load, the time delay and packet loss rate are very large. With ACO, RTT is slightly improved compared to RR, while packet loss rate is significantly reduced as around 0.28% (*h*_2_)~0.38% (*h*_8_). This indicates the effectiveness of the use of the ACO algorithm in distributing the load. Notice that the proposed G-ACO algorithm is very effective for LB as the time delay and packet loss rate are almost the same regardless of the destinations. In particular, the packet loss rate is as low as 0.13% (*h*_2_)~0.20% (*h*_8_).

Table 6 lists the path from *h*_1_ to other hosts. Observe that same paths are taken by the RR and ACO scheme, while the proposed G-ACO scheme more widely distributes the packets throughout the network than the other schemes. With RR and ACO, seven nodes (*e*_7_, *a*_3_, *e*_8_, *c*_2_, *a*_6_, *e*_9_. *e*_10_) are visited for the seven destinations, while nine nodes (*e*_7_, *a*_4_, *e*_8_, *c*_1_, *a*_6_, *e*_9_, *a*_3_, *e*_9_, *e*_10_) are visited with the proposed G-ACO scheme. With the involvement of two additional nodes, the packets can be more evenly distributed, which allows the enhancement in the performance of networking.

**Figure 11:**
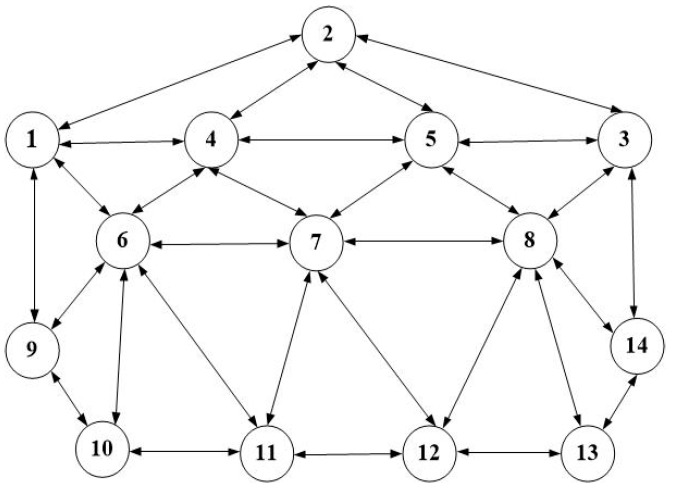
The 14-node topology.

**Figure 12:**
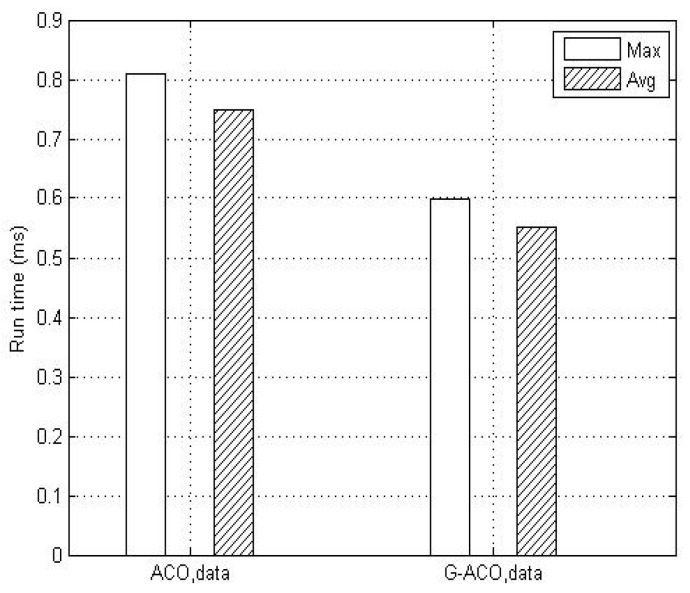
The comparison of running time with the 14-node topology.

For more comprehensive evaluation, the schemes are also tested with another topology of 14 nodes of Figure 11 [4]. Here, *srcNode* = 1 and *dstNode* = 13, and file data are transmitted at the rate of 1 Mbps. Notice from Figure 12 that the proposed G-ACO scheme consistently reduces the run time compared to the general ACO scheme.

## 5. Conclusions

In this paper we have presented the G-ACO scheme for LB of SDN. The proposed G-ACO scheme combines the selection, crossover and mutation operation of GA with the ACO algorithm to enhance the path search speed and the capability of searching an optimal path. LB is a very important issue for SDN due to the decoupling of the control plane and data forwarding plane. By implementing the proposed scheme into the LB module of OpenDayLight controller, an experiment was carried out with two networks topologies. The proposed scheme was also compared with the RR algorithm and ACO algorithm. The simulation results show that the proposed G-ACO scheme significantly improves the transmission time and packet loss rate. This is due to the effectiveness of the proposed scheme in searching the path and LB.

In the proposed G-ACO scheme, several factors were determined empirically, which significantly affect the overall performance. In the future, analytical models will be developed by which the value of the factors can be properly determined. Also, more comprehensive experiments will be conducted to investigate the relationships between the design parameters of SDN, and thereby the management of entire network can be finely tuned according to various operational conditions.

## Figures and Tables

**Table 1:** The fields of a flow entry in the flow table.

Field	Description
Match	Port, packet header, and metadata forwarded from the previous flow table
Priority	Matching precedence of the entry
Counter	Statistics for matching the packets
Instruction	Action or pipeline processing
Timeout	Maximum effective time or free time before the entry is overdue
Cookie	Opaque data sent by the OpenFlow controller

**Table 2:** The specification of the PCs.

	PC_1_	PC_2_
OS	Windows 10	Ubuntu 16.04
Virtual Machine	VMware Workstation 12 (OS: Ubuntu 16.04)	None
CPU	i3-4150	i3-4350
RAM	8G	8G
Hard Disk	500G	500G

**Table 3:** The number of iterations with different *ρ*.

Pheromone Volatilization Factor (*ρ*)	Iterations
0.1	3
0.3	7
0.5	8
0.7	13
0.9	28

**Table 4:** The number iterations taken to find an optimal path.

*α*	*B*	Iterations
0.1	0.1	33
0.1	0.5	17
0.5	0.5	8
1	2	7
3	7	3
5	9	2

**Table 5:** The number of ants and iterations.

*M*	Iterations
2	20
4	12
6	10
8	9
10	2

**Table 6:** The selected path with the three schemes.

Destination	RR & ACO	G-ACO
*h*_2_	*h*_1_-*e*_7_-*h*_2_	*h*_1_-*e*_7_-*h*_2_
*h*_3_	*h*_1_-*e*_7_-*a*_3_-*e*_8_-*h*_3_	*h*_1_-*e*_7_-*a*_4_-*e*_8_-*h*_3_
*h*_4_	*h*_1_-*e*_7_-*a*_3_-*e*_8_-*h*_4_	*h*_1_-*e*_7_-*a*_4_-*e*_8_-*h*_4_
*h*_5_	*h*_1_-*e*_7_-*a*_3_-*c*_2_-*a*_6_-*e*_9_-*h*_5_	*h*_1_-*e*_7_-*a*_4_-*c*_1_-*a*_6_-*e*_9_-*h*_5_
*h*_6_	*h*_1_-*e*_7_-*a*_3_-*c*_2_-*a*_6_-*e*_9_-*h*_6_	*h*_1_-*e*_7_-*a*_4_-*c*_1_-*a*_6_-*e*_9_-*h*_6_
*h*_7_	*h*_1_-*e*_7_-*a*_3_-*c*_2_-*a*_6_-*e*_10_-*h*_7_	*h*_1_-*e*_7_-*a*_3_-*c*_1_-*a*_6_-*e*_10_-*h*_7_
*h*_8_	*h*_1_-*e*_7_-*a*_3_-*c*_2_-*a*_6_-*e*_10_-*h*_8_	*h*_1_-*e*_7_-*a*_3_-*c*_1_-*a*_6_-*e*_10_-*h*_8_
